# Born to Win: Reproductive Health Policy and Urban Education Returns in China

**DOI:** 10.3389/fpubh.2022.807794

**Published:** 2022-03-03

**Authors:** Qing Gao, Xiu-Xiu Ren, Wei-Lan Yan, Ai-Hua Wang, Wei-Hua Zhang

**Affiliations:** ^1^Key Laboratory of Environment Change and Resources Use in Beibu Gulf Ministry of Education, Nanning Normal University, Nanning, China; ^2^Graduate School, Nanning Normal University, Nanning, China; ^3^College of Economics and Management, Nanning Normal University, Nanning, China; ^4^School of International Economics and Trade, Guangxi University of Foreign Languages, Nanning, China; ^5^School of Economics, Guangxi University, Nanning, China; ^6^Guangxi Development and Reform Commission, Nanning, China

**Keywords:** born to win, residents' reproductive health policy, regression discontinuity model, return on education, intermediary effect

## Abstract

This study explores the relationship between China's reproductive health policy and the rate of return on urban education by using fixed-effect, mediating-effect, and breakpoint regression models. The authors study the impact of China's reproductive health policy on individual years of education, income, return on education, the impact of reproductive health policy on population health, and overall human development. The empirical results show that the implementation of China's reproductive health policy can improve the rate of return on urban education and increase the average length of education of urban residents by 0.29 years. Under the breakpoint regression model, the educational return rate of urban residents is approximately 12.2% higher than the ordinary least squares (OLS) estimate. China's reproductive health policy has significantly promoted the return rate of residents' education and simultaneously effectively promoted the income of urban residents. With the development of the economy, the government should properly adjust reproductive health policies and improve the population health rate so that the adjustment of reproductive health policies can comprehensively promote the health of residents of all ages. The overall relaxation of family restrictions on children's human capital investment will likely reduce the negative impact of the overall relaxation of family planning, not only improving the health of the population but also contributing to sustainable economic development.

## Introduction

The purpose of this study is to explore whether the implementation of the one-child policy will affect the rate of return on education for urban residents and if so, whether it will “promote” or “inhibit” this rate of return. Since the cultural reform and societal opening over 40 years ago include rapid economic development, the population has grown substantially with population health becoming threatened. Population health is a major problem that restricts China's comprehensive, coordinated, and sustainable development. In 1980, problems arising from family planning policy, economic development, a lack of universal education, the overall quality of the population, and the health of the population were alleviated. The “one-child” policy in particular greatly contributed to improving population health. However, as the population has aged, a two-child policy was introduced in November 2011. The “three-child” policy will be implemented on May 31, 2021. The implementation of the policy has a strong role in promoting the birth quality of the next generation and promoting the long-term balanced and healthy development of population quality by improving the quality of prenatal and postnatal care, health status, and education status. Research on reproductive health has attracted much attention. What is the impact of reproductive health policy on the rate of return of education? The rate of return on education, which measures the impact of an individual's education level on his income, is an important issue in education economics, labor economics, and health economics. China is in a period of rapid development of its economy and education system, and as economic transformation and education reform continue to deepen, the income gap of residents has been increasing. Education is an important method of human capital investment and an important factor of economic development. High-quality human capital development has a positive effect on social population health and is a key element of social progress.

Education is closely related to income level. Some studies have pointed out the heterogeneous effect of education investment on income level and the income gap (1). The development of education can promote the adoption of modern technology and improve individual income levels, and education development also has a positive effect on the improvement of social health (2). As a result of the reproductive health policy, the per capita income level increased. Simultaneously, China's illiteracy rate fell rapidly, to 4.08 percent in 2010, while on the other hand, the average number of years of education received by the population over the age of 6 increased from 5.3 years in 1982 to 9.3 years in 2013. The survey data in 2021 showed that the average number of years of education received by the population over the age of 15 increased from 9.08 to 9.91 years, and the illiteracy rate decreased from 4.08% to 2.67%. Meanwhile, from 1982 to 2017, China's gross domestic product (GDP) grew by 10% in real terms, and the size of the GDP increased nearly 20-fold. Research on the effect of reproductive health policy on the rate of return on education in China is relatively weak. It is important to reasonably control the impact of reproductive health policy on the rate of return on education and to master the changing trend of the rate of return on education. It is also of great practical significance to accelerate the accumulation of human capital, improve the efficiency of education resource allocation, rationally formulate education development policy, and reproductive health policy, promote the marketization process of the labor market and optimize individual education decision-making.

According to the Report on the Development of Chinese People's Livelihood 2016, released by the China Social Science Survey Center of Peking University in 2014, the per capita income of families in the bottom 5% of rural areas was 600 yuan and that of families in the top 5% of urban areas was 53,300 yuan, a gap of 88.8 times between the two poles. If the gap between the rich and poor continues to widen, social conflicts are more likely to intensify and sustainable economic development will be hindered. China's Gini coefficient stood at 0.465 in 2016, still above 0.4, the warning line for the gap between rich and poor. Therefore, studying the influencing factors of per capita family income and discussing how to improve the level of education and health of the family to increase family income is of great research value for the realization of an all-around well-off society. A healthy yield of 36.3% is significant at the 1% level, with “healthy” referring to being in good physical condition. People who are in good physical health tend to have more energy, work longer hours, be more productive and creative, and thus have a higher level of income. In addition, healthy individuals tend to have a good attitude in work and life and are more willing to invest in their human capital, which is conducive to the long-term development of personal careers. Overall, good health is an important factor in improving individual income.

This study mainly focuses on two aspects. First, this study estimates the impact of the implementation of the family planning policy on the average number of years of education. Second, this study estimates the rate of return on education using data from the China General Social Survey (CGSS) in 2008 and 2010. The contributions of this study are as follows: On the one hand, this study evaluates the impact of the implementation of China's reproductive health policy on educational returns. By means of breakpoint regression and using RD parameter estimation and non-parameter estimation under quasi-natural experimental conditions, the causal effect of reproductive health policy on per capita schooling years is studied, and possible problems of endogeneity are avoided. On the other hand, through regression discontinuity design (RD) regression, we confirm that the education “breakpoint” before and after the cut-off is 0.29 years; that is, the implementation of compulsory education law quickly leads to a jump of 0.29 years of education per capita for the people affected by the policy. The RD estimation results show that the educational return rate in China is 12.2%, which is significantly higher than the OLS estimation results. OLS estimation of educational returns has the problem of underestimation.

The rest of the article is structured as follows. The literature review section reviews the existing literature. The model of reproductive health policy and urban education return rate describes the theoretical mechanism of reproductive health policy and education return rate. The data and econometric model section describe the micro data in this study. The method section introduces the empirical method. The empirical results show the research results. The conclusion section outlines the conclusions from the study.

## Literature Review

The classic model of educational return rate is the Mincer equation, which takes the logarithmic form of income as the explained variable, including years of education, work experience and the square term of work experience, and other variables that affect individual income (such as sex, ethnicity, marital status, and health status). According to the actual situation, the Mincer equation is improved to varying degrees, such as adding more control variables or using instrumental variables on the basis of the standard Mincer equation to reduce the estimated deviation caused by missing variables and measurement errors (3). In addition, scholars relaxed the assumption of individual homogeneity and corrected the sample selection bias. David Card et al. (4) provided a good summary of studies on the educational return rate mainly in the United States.

Analyses of the educational rate of return models. Starting from the estimation of the rate of return on education, relevant representative studies discussed the changing trend in the rate of return on education, the factors influencing the rate of return on education, the impact of the rate of return on the income gap and labor mobility, and the heterogeneous rate of return on education in different regions, industries, school segments, and groups (5). Many scholars estimate the dynamic change in the rate of return of urban education in China on the basis of controlling individual ability and family characteristics by using sample survey data and the Mincer equation. Some scholars have estimated the rate of return on higher education in China and tested heterogeneous educational returns (6). Some scholars focus on the regional heterogeneity in the educational return rate. In addition, some scholars pay attention to the impact of city size and local government education expenditure on the educational rate of return (7). Some scholars have also used quantile regression to estimate and discuss the educational return rate and its relationship with the income gap in China (8). In the econometric analysis of the education economy and labor economy, there are many methods to analyze the rate of return of education, such as OLS estimation (9, 10). This has many methods, but the ordinary OLS estimation method is used to estimate the rate of return on education. The deviation of the rate of return on education caused by endogeneity problems is too large to be ignored, and it overlooks the correlation between individual ability and individual education level. Many scholars used twin data to control endogenous variables (11) and found that the measurement error of educational return estimated based on twin data was higher than that of the OLS estimation method, but the estimation of educational return was not accurate enough.

Analyses based on endogeneity. Owing to the endogeneity of education, when estimating the rate of return on education, we consider only the impact of observable factors while ignoring non-observable factors, such as personal ability. In real life, we will find that two individuals with the same characteristics have received the same education and have the same work experience. There are three ways to solve the endogeneity of education: one is to find proxy variables of ability and add them into the Mincer equation. Controlling the variables with other observable variables makes education and error terms irrelevant. The second way to solve the endogeneity of education is to use the instrumental variable method to estimate the rate of return on education. This method is to find a tool that is highly correlated with education but not with the error term to eliminate endogeneity. However, it is difficult to find a satisfactory instrumental variable, and it is easy to cause the problem of weak instrumental variables. The third way to solve the endogeneity of education is to use the fixed-effect method. Twin data are used for empirical analysis, but this method is demanding and difficult to implement. Many scholars use different methods to solve the endogeneity problem caused by estimation methods. At present, most studies adopt the instrumental variable method. Heckman and Li (12) used 2000 China urban household income and expending survey (CUHIES) data to analyze the heterogeneity caused by variables and concluded that the educational return rate of Chinese colleges and universities is approximately 11%. Chen and Hamori (13) used the data of 2004 and 2006 China health and nutrition survey (CHNS) to calculate the educational return rate of males and females in urban China by using OLS and instrumental variable (IV) estimation methods, respectively. The results showed that the OLS calculated result was 8.06% for males and 7.67% for females. The results estimated by IV were 12.61% for men and 14.47% for women. After adjusting for sample selection bias for women, the return on education for women was 21.5%.

Analyses of the impact of reproductive health policy on education level. To reduce the burden in the field of education, we should strengthen the reform in the field of education and promote the quality and balanced development of compulsory education. The implementation regulations promulgated by different provinces vary over time. Due to the difference in time and the region of the promulgation of the reproductive health policy, the educational return rate will be affected by the policy differently. Research on the relationship between reproductive health policy and educational well-being (14), on the impact of the adjustment of reproductive health policy on the allocation of urban basic education resources (15), on the equality of reproductive health policy on gender education (16) and others have analyzed the impact of reproductive health policy on education, but all were based on the macro level. Juan Liao (17) studied the heterogeneity analysis of educational return, saying that the traditional Mincer equation method was no longer applicable and adopted a new model nonparametric estimation method (partial linear model) to study the problem of educational return. Sample regression and stratification models, or multilevel models, are also commonly used to study the heterogeneity of educational returns by controlling for other factors that affect an individual's educational level. If the fertility level does not increase substantially in the future, the sustainable development of the social economy will be challenged (18). Based on a sustained drop in fertility and a simple relaxation of the control policy's effect being suboptimal, the family birth level is lower than the fertility policy meant to cope with an aging population and change the status quo of the current low fertility rates. Therefore, building a family-friendly society has increasingly become the consensus of people from all walks of life, and increased spending on education as a birth gripper optimization plays an important role in the building of the social policy. So, what is the effect of the reproductive health policy on the education level?

In summary, the research results based on different countries and different perspectives show that reproductive health policy has a significant effect on promoting the rate of return on urban education, and the estimation results using the RD estimation method are more characteristic of random experiments compared with IV estimation (19). In addition, RD estimation can estimate the local average processing effect by controlling the window width. The RD estimation method can control the estimation deviation of educational returns caused by endogeneity problems and can accurately estimate the effect of the policy system on educational returns. This is important for estimating the return on education in China.

## Reproductive Health Policy and Urban Education Rate of Return Model

### The Relationship Between Reproductive Health Policy and Educational Return Rate

In the early days of the founding of New China, facing the population base, China's reproductive health policy mainly included seven stages, as seen in Table 1.

**Table 1:** The history of China's reproductive health policy.

**Stage**	**Year**	**Content**
The first stage	In 1949–1961	The concept of family planning was put forward, many documents put forward the problem of large population base.
The second stage	In 1962–1978	The family planning policy was put forward.
The third stage	In 1979–1991	Family planning became a basic state policy and the “one child” policy was implemented.
The fourth stage	In 1992–2000	The family planning policy is sound, and the population is under certain control.
The fifth stage	In 2001–2012	The birth rate drops gradually, the population problem is solved, and the problem of aging appears.
The sixth stage	In 2013–2020	The two-child policy has been relaxed to address the growing problem of an aging population.
The seventh stage	In May 31, 2021	We has been implemented a three-child policy?

The course of China's one-child policy indicates that China's population control has experienced a “loose and tight to loose” tortuous development process (20), thereby impacting population health. The implementation of the “one-child” policy reduced the birth rate, and the decrease of the birth rate and the improvement of the health level of the population lead to the trend that the annual total income of individuals decreases first and then increases, so there is a breakpoint of downward jump. The declining trend in individual annual total income makes the number of years of education decrease substantially at the beginning and then increase. This indicates that the implementation of reproductive health policy affects the income and average length of education of the observed individuals.

Breakpoint regression can use the discontinuous behavior caused by exogenous policy impact to estimate the effect of policy implementation and effectively identify the causal relationship between variables with realistic constraints. Since the 1990s, breakpoint regression has gradually been applied to many economic fields, such as the labor economy, environmental economy, and regional economy. However, at present, few studies use breakpoint regression design to estimate the rate of return on education and the effect of reproductive health policy. According to the breakpoint, the basic idea of a regression design without implementing the one-child policy, individual education fixed number of years, and the probability of receiving higher education should be a smooth change along with the age. If it is found that the fixed number of years of the individuals affected by education and income breakpoints before and after policy implementation, it may be assumed that these differences are brought on by exogenous policy factors. Therefore, breakpoint regression can identify the causal influence between education and individual income by using the change in years of education and the probability of receiving higher education caused by reproductive health policy. Breakpoint regression is a relatively effective way to measure the effect of the policy. Education is the foundation of a country, particularly a strong country. The prosperity of a country cannot be achieved without education. The reproductive health policy is a basic state policy of China. At present, many studies have examined the relationship between the reproductive health policy and education, and some studies have explored the negative relationship between the number of children in a family and education level through empirical research (21), and there is no direct and significant relationship between the two (22). The birth order of children is the main factor influencing education level; some studies have found that there is a positive relationship between the two. Shige Song (23) believed that the two-child policy significantly increased the enrolment rate of the first child. Based on the above controversy, Hypothesis 1 is proposed.

Hypothesis 1: The reproductive health policy has a significant promoting effect on the educational rate of return.

### The Mediating Effect of Consumer Spending

The one-child policy affects household consumption expenditures, including expenditures on food, clothing, housing, household goods and services, transportation and communication, education, culture and entertainment, and medical care. In the early days of the founding of the People's Republic of China, population health was a major problem restricting China's comprehensive, coordinated, and sustainable development and was a key factor affecting economic development. Educational consumption is closely related to income distribution, which determines the possible level of educational consumption (24). From the macro point of view, the income distribution of a country or region determines the educational consumption level that the country or region may achieve. From the micro point of view, the expenditure a family may make on education consumption is affected by the income level of the family. The implementation of family planning effectively relieved the pressure on resources and the population, easing population health problems and reducing the consumption of the new population. To accelerate national capital accumulation, the nation has accumulated funds for education and more people have additional education opportunities and a better quality of education, which is beneficial for improving the quality of the population of China, thus improving the return on education. The improvement in the rate of return on education, on the one hand, can increase family income while relatively reducing family consumption, so that the main members of the family can spare more time and energy to develop the family income and education to ensure the health and happiness of the family and social stability. On the other hand, the improvement in the rate of return on education can alleviate the contradiction between man and land, optimizing the allocation of resources and improving the per capita level of resources. This also improves the quality of the population, the purpose of which is improving the rate of return on education. Based on the above analysis, the implementation of the reproductive health policy can have a mediating effect on the rate of return of education through consumption expenditure. Therefore, Hypothesis 2 is proposed:

Hypothesis 2: The implementation of the reproductive health policy can affect the change in consumption expenditure in a positive direction. An increase in consumer spending can increase the rate of return on education.

### Mediating Effect of Housing Area

To a certain extent, the housing area of a family reflects the economic income of a family. The implementation of the family planning policy will increase the per capita housing area of families and release housing demand. The excess housing area can be used for investment, which will increase household savings and increase the amount of investment in education. At the same time, the improvement in the education level increases family housing areas (25). Families with large numbers of children have larger housing areas but smaller per capita housing areas with poorer housing quality. Therefore, the implementation of the family planning policy increases the per capita housing area and leads to better housing quality and better health. On the one hand, housing area can be adjusted automatically through the adjustment of the family planning policy to affect one's education level, which can be used as housing investment or education investment. On the other hand, the implementation of a reproductive health policy will affect the per capita income of the family and the size of the housing area of the family, thus affecting the contribution rate of education return. Therefore, Hypothesis 3 is proposed:

Hypothesis 3: The implementation of the reproductive health policy can affect the size of housing areas in a positive direction. An increase in housing area can increase the rate of return on education.

## Data

### Data Sources

The data used in this study came from the China General Social Survey (CGSS) conducted by the National Bureau of Statistics of China in 2008 and 2010. The sample includes 24 provinces, including Shanghai, Beijing, Jilin, Tianjin, Fujian, Anhui, Guangdong, Guangxi, Hebei, Henan, Guizhou, Yunnan, Hubei, Hunan, Heilongjiang, Liaoning, Jiangsu, Jiangxi, Zhejiang, Chongqing, Inner Mongolia, Shanxi, Shaanxi, and Liaoning. From the perspective of China's economic development level, there is an obvious gap.

Since the research is on the impact of China's one-child policy on the rate of return on education, to ensure the accuracy of the data analysis, school students, retirees, and household workers were excluded. Mainly studied was the effect of the “one-child” policy on the rate of return on education. Because of the “one-child” policy, which was implemented in September 1980, individuals born after that year (sample size is 12,875) were now of legal working age at 16 and therefore affected by the reproductive health policy. The data were divided into eight educational levels: never attended school (0 years), primary school (6 years), junior high school (9 years), senior high school (12 years), technical secondary school (13 years), junior college (15 years), bachelor's degree (16 years), and master's degree (19 years). On the basis of urban and rural household survey data, we divided urban and rural residents into separate datasets to improve the accuracy of the data. At the same time, we also took into account the individual screen out and transformation that occur in the transition from rural to urban areas. After the above processing, we obtained 4,678 rural samples and 6,485 urban samples.

### Set Variables

#### Explained Variables

The explained variable is the natural logarithm of the annual total income of individuals using the logarithm data of the annual total income of CGSS respondents.

#### Core Explanatory Variables

Core explanatory variables include years of schooling and reproductive health policy. Among them, the number of years of education refers to the number of years of education an individual receives. The reproductive health policy is the number of children.

#### Intermediary Variables

The mediating variables include consumption expenditure and housing area. The mediating variables are introduced to further explore the mechanism of reproductive health policy's rate of return on education.

#### Control Variables

The control variables are set by the Mincer equation, including work experience (exp), the square term of work experience (exp^2^), age, sex, ethnicity, marital status, health status, etc., as seen in Table 2.

**Table 2:** Description of variables.

**Variable symbol**	**The variable name**	**Variable meaning**
*lny*	Individual annual income	Natural logarithm of individual annual income (yuan)
*Policy*	One child policy	Individual families with one child or two children = 1; The rest of 0
*Edu*	Years of education	Never went to school; Primary level =6; Junior high = 9; High school = 12; Technical secondary school = 13; Associate degree = 15; Bachelor degree = 16; Graduate level = 19.
exp	Work experience	Work experience = year of questionnaire–year of birth–Years of education−6
exp^2^	Work experience squared	Work experience squared
*Gender*	Gender	Male = 1, female = 0
*National*	National	Han = 1; Other nationalities = 0
*Health*	Health	Healthy = 1, unhealthy = 0
*Hunyin*	Marital status	Married with spouse = 1; Other marital status = 0
*Year*	Time	In 2008 and 2010
*Cons*	Consumer spending	Household consumption expenses
*House*	Housing area	The floor area of a house

## Methodology

### OLS Estimation

To test the impact of reproductive health policy on the urban educational return rate, the following benchmark regression model was constructed. The extended equation of Mincer (26) was used to study the impact of reproductive health policy on the educational return rate.


(1) $$\begin{array}{l} ln\;y_{i}=\sigma +\beta_{1}edu_{it}+\beta_{2}fertility_{it}+\beta_{3}\exp_{it}+\beta_{4}{\exp^{2}}_{it}\\ \;+\theta X_{i}+\varepsilon_{i} \end{array}$$

where *lny*_*i*_ is the logarithmic form of individual annual total income, which is used as the explained variable. *edu*_*it*_ represents the education years of individual *i*, which is the explanatory variable of main concern. exp_*i*_ is the square term of *i*'s work experience and refers to the control variables, including *gender*, *marriage national*, and *health*.

### Mediating Effect

To examine the impact of reproductive health policy on the rate of return on education, the mediation effect theory in this paper was tested by the analytic hierarchy process (AHP) to examine the difference in consumption expenditure on the rate of only children of different individuals (27). Therefore, the mediation effect estimation model is established as follows:


(2) $$\begin{array}{l} cons_{it}=\eta_{0}+\eta_{1}fertility_{it}+\eta_{2}edu_{it}+\eta_{3}\exp_{it}+\eta_{4}{\exp^{2}}_{it}\\ \;+\eta_{5}X_{i}+\mu_{i}+\varepsilon_{it} \end{array}$$

(3) $$\begin{array}{l} house_{it}=\alpha_{0}+\alpha_{1}fertility_{it}+\alpha_{2}edu_{it}+\alpha_{3}\exp_{it}+\alpha_{4}{\exp^{2}}_{it}\\ \;+\alpha_{5}X_{i}+\nu_{i}+\varepsilon_{it} \end{array}$$

(4) $$\begin{array}{l} lny_{i}=\phi_{0}+\phi_{1}fertility_{it}+\phi_{2}edu_{it}+\phi_{3}cons_{it}+\phi_{4}house_{it}\\ \;+\phi_{5}\exp_{it}+\phi_{6}{\exp^{2}}_{it}+\theta X_{i}+\mu_{i}{+\nu_{i}+\varepsilon }_{i} \end{array}$$

In Formula (2), *cons*_*it*_ is consumption expenditure, and *house*_*it*_ is individual housing area. From the two aspects of consumption and savings (housing area), two different effects can be obtained through the test of Equations (2) and (3). The mediating effect can be divided into two stages: independent variable to the mediating variable and the mediating variable to the dependent variable. The mediating effect of consumer expenditure is η_1_ and that of housing area is α_1_.

### RD Estimation

Breakpoint regression methods can be divided into obvious breakpoint regression (SRD) and fuzzy breakpoint regression (FRD). With fuzzy breakpoints (28), the basic idea of regression design is whether resident I is affected by China's reproductive health policy or entirely depends on the value of driving variables on both sides of the breakpoint (29). Parameter estimation and non-parameter estimation can be used in the above estimation, and the parameter estimation method is adopted here. In the fuzzy RDD framework, the standard method of parameter estimation is 2SLS, and the specific equation is set as follows:


(5) $$\begin{array}{l} lny_{i}=\delta_{0}+\delta_{1}fertility_{it}+h\left(cutoff\right)+\;\mu_{i} \end{array}$$

(6) $$\begin{array}{l} edu_{i}=\beta_{0}+\beta_{1}fertility_{it}+g\left(cutoff\right)+\;\gamma_{i} \end{array}$$

The structural form of the education rate of return equation is:


(7) $$\begin{array}{l} lny_{i}=\vartheta_{0}+\alpha_{FRDD}edu_{it}+h\left(cutoff\right)+\;\varepsilon_{i} \end{array}$$

Here, *h*(•), *g*(•), and *f*(•) are smooth functions of driving variables. In practice, *h*(•), *g*(•), and *f*(•) are defined as low-order polynomial models on both sides of (*cutoff*), and the polynomial models on both sides are assumed to have the same order and different slopes. The estimated coefficient of the instrumental variable of the educational return rate in Equation (7) is the ratio of the estimated coefficient of simplified Equation (5) and Equation (6), α_*FRDD*_ = δ_1_/β_1_. According to the implementation of family planning since 1980, the education status of people born after 1980 is affected by the implementation of China's family planning policy. We call them the treatment group, and the corresponding group born before 1980 is called the control group. If the individual was born after 1980, the dummy variable of the reproductive health policy is defined as 1; otherwise, it is 0.

## Empirical Results

### Descriptive Statistics

As seen from Table 3, the standard deviation of the logarithm of individual annual total income is 1.186, indicating that there is a large income gap between individuals, which may be a regional gap. The average number of years of education of individuals is 9.27, and the variance is 4.23, indicating the large difference in the number of years of education of the observed samples. The survey data also identified other variables related to individual income that reflect individual characteristics, including ethnicity, marital status, sex, and health status.

**Table 3:** Sample size and distribution: 2007–2009.

**Variable**	**The variable name**	**obs**	**Mean**	**Std.Dev**
*lny*	Annual personal gross income	10,214	9.194	1.186
*Policy*	Number of children	12,819	1.622	1.247
*Edu*	The number of years an individual has been in education	12,864	9.256	4.288
*Gender*	Gender	12,875	0.484	0.5
*Birth*	Year of birth	12,875	1,967.799	11.488
*exp*	Work experience	12,836	24.129	12.194
*exp^2^*	Work experience squared	12,838	730.767	603.729
*National*	National	12,863	0.916	0.278
*Health*	Health	12,875	0.872	0.334
*Hunyin*	Marital status	11,570	0.946	0.226
*House*	Housing floor area	12,744	111.957	95.438
*Cons*	Consumer spending	12,117	5,988.156	19,953.275
*Province*	Provinces	12,875	15.452	7.965

### Return on Education: OLS Estimation

OLS was first used to estimate the rate of return on education. Table 4 shows the OLS estimation results of the impact of years of education on all samples. Column (1) is defined according to the basic elements of the classic Mincer equation, and the regression results show that the return on education is 14.2%. Control variables were added from Model (2) to Model (5), and dummy variables of sex, ethnicity, health status, and marriage were added. In Column (2), a dummy variable of sex was added, and the results showed that the males' income was approximately 39.3% higher than the females' income. In Column (3), the dummy variable of ethnic group was added. According to the results, ethnic group has no influence on individual annual total income. The dummy variable of individual health status was added in Column (4). The results show that the education level of healthy individuals is 42.95% higher than that of nonhealthy individuals, it shows that the good health of residents is conducive to the improvement of educational returns, the so-called health is the capital of the revolution. The dummy variable of individual marital status is added in Column (5). From the results, marital status has no significant influence on an individual's annual total income. The estimated results of Column (6) indicate that the rate of return on education in rural areas is 5.31%, and the estimated results of Column (7) indicate that the rate of return on education in urban areas is 11.72%. According to the results in Columns (6) and (7), the educational return rate for urban residents is significantly higher than that for rural residents, which means that there is a Matthew effect between urban and rural areas, where the poorer the residents in the countryside are, the richer the residents in cities are. Therefore, considering the relationship between reproductive health policy and the rate of return on education, what kind of impact will the reproductive health policy have on the rate of return on urban education? The next step is to introduce intermediary variables to analyze reproductive health policies.

**Table 4:** Educational returns estimated by OLS.

***Variable***	**(1)**	**(2)**	**(3)**	**(4)**	**(5)**	**(6)**	**(7)**
	**All**	**All**	**All**	**All**	**All**	农村	城市
*Edu*	0.1466*** (0.0026)	0.1379*** (0.0027)	0.1361*** (0.0027)	0.1306*** (0.0027)	0.1261*** (0.0028)	0.0531*** (0.0048)	0.1172*** (0.0037)
*Policy*	−0.0555*** (0.0080)	−0.0545*** (0.0078)	0.0530*** (0.0078)	0.0515*** (0.0077)	0.0661*** (0.0082)	0.0573*** (0.0123)	−0.0183** (0.0100)
*lnexp*	−0.0197*** (0.0021)	−0.0224*** (0.0021)	0.0230*** (0.0021)	0.0194*** (0.0021)	0.0161*** (0.0027)	0.0177*** (0.0045)	0.0140*** (0.0031)
lnexp^2^	0.1188*** (0.0160)	0.1250*** (0.0161)	0.1276*** (0.0160)	0.1125*** (0.0159)	0.0696** (0.0244)	0.0444 (0.0487)	0.0743** (0.0272)
*Gender*		0.3644 (0.0192)	0.3634 (0.0192)	0.3426 (0.0191)	0.3781 (0.0203)	0.5495 (0.0335)	0.3629 (0.0234)
*National*			0.2577*** (0.0350)	0.2540*** (0.0347)	0.2568*** (0.0364)	0.1674*** (0.0472)	0.2040*** (0.0523)
*Health*				0.4300*** (0.0320)	0.4323*** (0.0324)	0.3808*** (0.0391)	0.3603*** (0.0497)
*Hunyin*					−0.0706 (0.0437)	0.0973 (0.0656)	−0.0342 (0.0531)
_cons	7.6813*** (0.0653)	7.5948*** (0.0661)	7.3731*** (0.0730)	7.0623*** (0.0766)	7.3588*** (0.1145)	7.6433*** (0.2211)	7.6102*** (0.1401)
*N*	10,132	10,132	10,125	10,125	9,226	4,030	5,196
*R* ^2^	0.3347	0.3575	0.3611	0.3743	0.3705	0.2123	0.3119
F	1,311.5590	1,126.7212	952.5378	850.1259	686.3687	144.6915	253.2361
p	0.0000	0.0000	0.0000	0.0000	0.0000	0.0000	0.0000

***, **, * distribution means that statistics with significance levels of 1%, 5%, and 10% can be passed.

### Mediating Effect Test

Hausmann's test results showed that the F value was 27.51 and the *P* value was 0.0003, indicating that the estimated results had a significant fixed effect. The results are shown in Table 5. In Column (8), there is a significant negative relationship between reproductive health policy and consumption expenditure, but the regression coefficient of years of education on consumption expenditure is significantly positive, and Hypothesis 2 is not valid. In Column (10), there is a significant positive relationship between reproductive health policy and housing area, but the regression coefficient of years of education on housing area is significantly positive, indicating that an increase in housing area can improve the rate of return on education. Hypothesis 3 is valid. Columns (9) and (11) are the results of taking consumption expenditure and housing area as control variables, respectively, and the years of education are both positive and significant. When the mediating variables are controlled, the regression coefficient between the independent variable and the individual's annual total income is still significant, indicating that consumer expenditure and housing area have a partially mediating effect on the reproductive health policy, years of education, and rate of return on education.

**Table 5:** Regression results of mediating effects.

	**(8)**	**(9)**	**(10)**	**(11)**	**(12)**
	**Consumer spending**	**All**	**Housing area**	**All**	**All**
*Policy*	−0.0944*** (0.0106)	−0.0454*** (0.0079)	0.0488*** (0.0049)	−0.0655*** (0.0082)	−0.0436*** (0.0079)
*Edu*	0.0573*** (0.0035)	0.1113*** (0.0027)	0.0144*** (0.0015)	0.1272*** (0.0028)	0.1111*** (0.0027)
*lnexp*	0.0315 (0.0541)	0.0479 (0.0056)	0.1840 (0.812)	0.0584 (0.0062)	0.0691 (0.251)
lnexp^2^	0.0275* (0.0125)	−0.0912*** (0.0097)	−0.0026 (0.0060)	−0.0788*** (0.0099)	−0.0913*** (0.0097)
*Gender*	−0.0248 (0.0255)	0.3679*** (0.0196)	0.0080 (0.0118)	0.3751*** (0.0203)	0.3691*** (0.0197)
*national*	0.0401 (0.0447)	0.2335*** (0.0350)	−0.0304 (0.0207)	0.2430*** (0.0364)	0.2276*** (0.0350)
*health*	0.2124*** (0.0411)	0.4091*** (0.0314)	0.0605*** (0.0168)	0.4548*** (0.0323)	0.4128*** (0.0314)
*hunyin*	−0.0027 (0.0581)	−0.0403 (0.0428)	0.2276*** (0.0258)	−0.0316 (0.0442)	−0.0300 (0.0432)
*lncons*		0.2407*** (0.0078)			0.2374*** (0.0078)
*lnhouse*				−0.0564*** (0.0163)	−0.0367* (0.0157)
_cons	7.1648*** (0.1238)	6.1621*** (0.1061)	4.3504*** (0.0565)	8.0781*** (0.1191)	6.3469*** (0.1246)
*N*	10,800	8,761	11,391	9,162	8,705
*R* ^2^	0.0514	0.4337	0.0282	0.3725	0.4345
F	79.8765	842.0963	50.3256	684.9616	742.5168
p	0.0000	0.0000	0.0000	0.0000	0.0000

***, ^**^, ^*^ distribution means that statistics with significance levels of 1%, 5%, and 10% can be passed.

### Educational Returns: RD Estimates

#### LATE Implementation of Reproductive Health Policy

**Figure 1:**
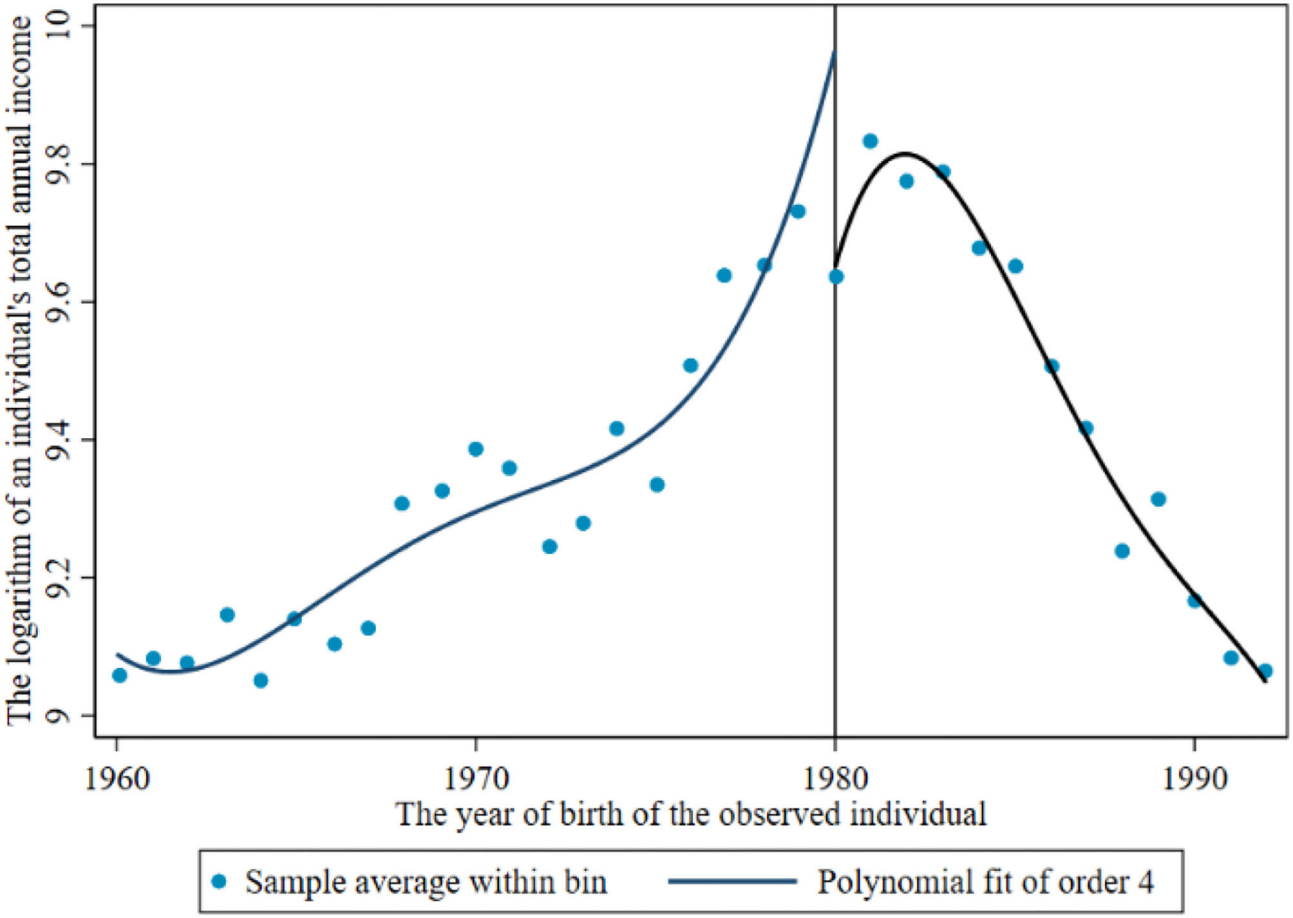
Income breakpoint of rural residents before and after the implementation of the reproductive health policy.

**Figure 2:**
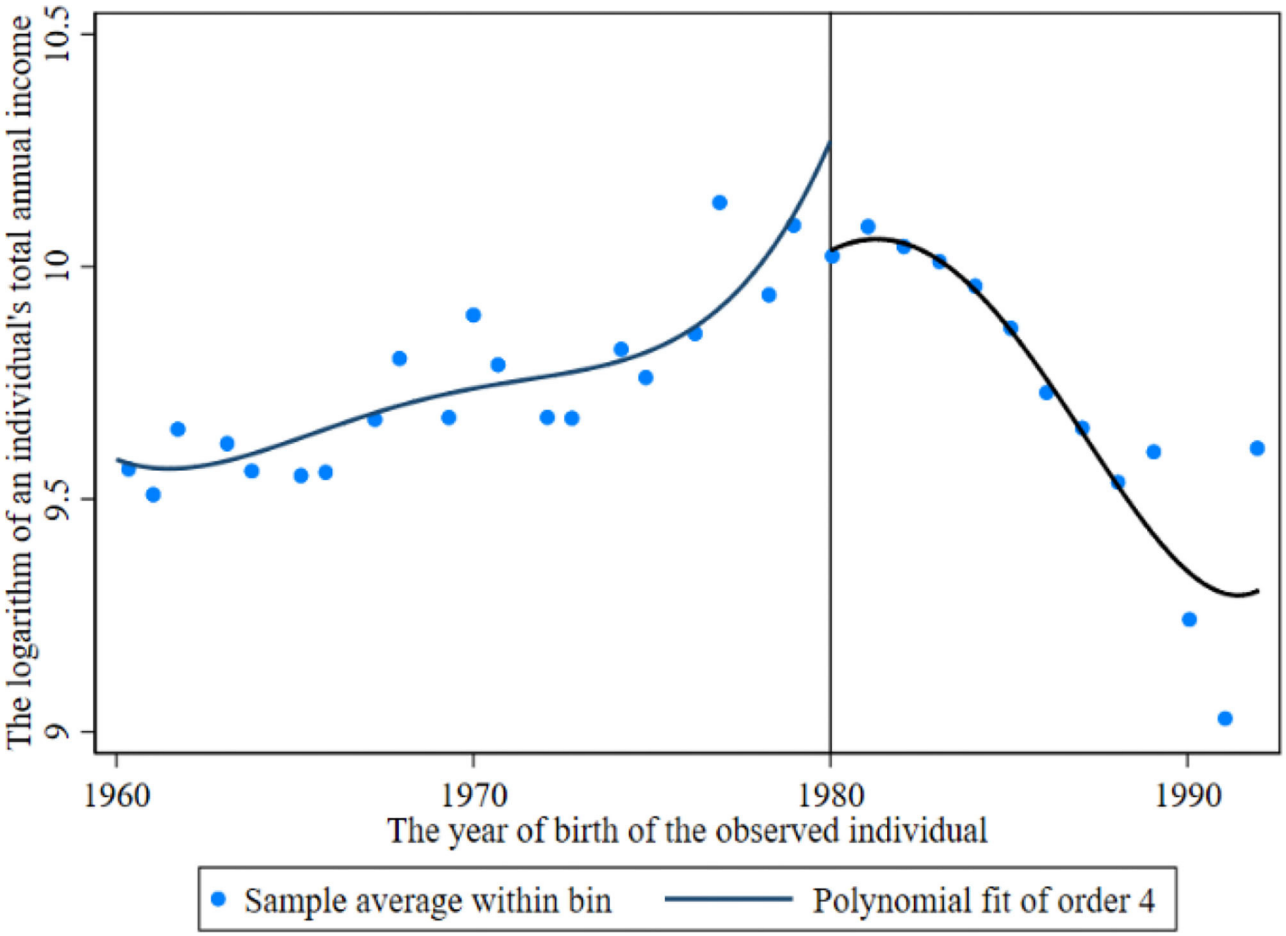
Income breakpoint of urban residents before and after the implementation of the reproductive health policy.

Before the return of the breakpoint, the breakpoint caused by the reproductive health policy is observed. Figures 1, 2 show the income breakpoints of rural and urban residents before and after the implementation of the family planning policy. At the breakpoint, we find that there is an obvious “jump”. As China's reproductive health policy has undergone six stages of change (30), population control has been loosened first and then tightened again, and there is an obvious change trend in the breakpoint chart with the degree of the loosening of policy implementation. The educational return rate of urban residents was higher than that of rural residents before and after the implementation of the policy, and the annual total income of individuals jumped between 10.2 and 12.3 after 1980. The reasons for this phenomenon may be as follows: the defect of the “omission” of survey data, which may miss some individuals who are receiving education, or the impact of the urban-rural income gap.

**Figure 3:**
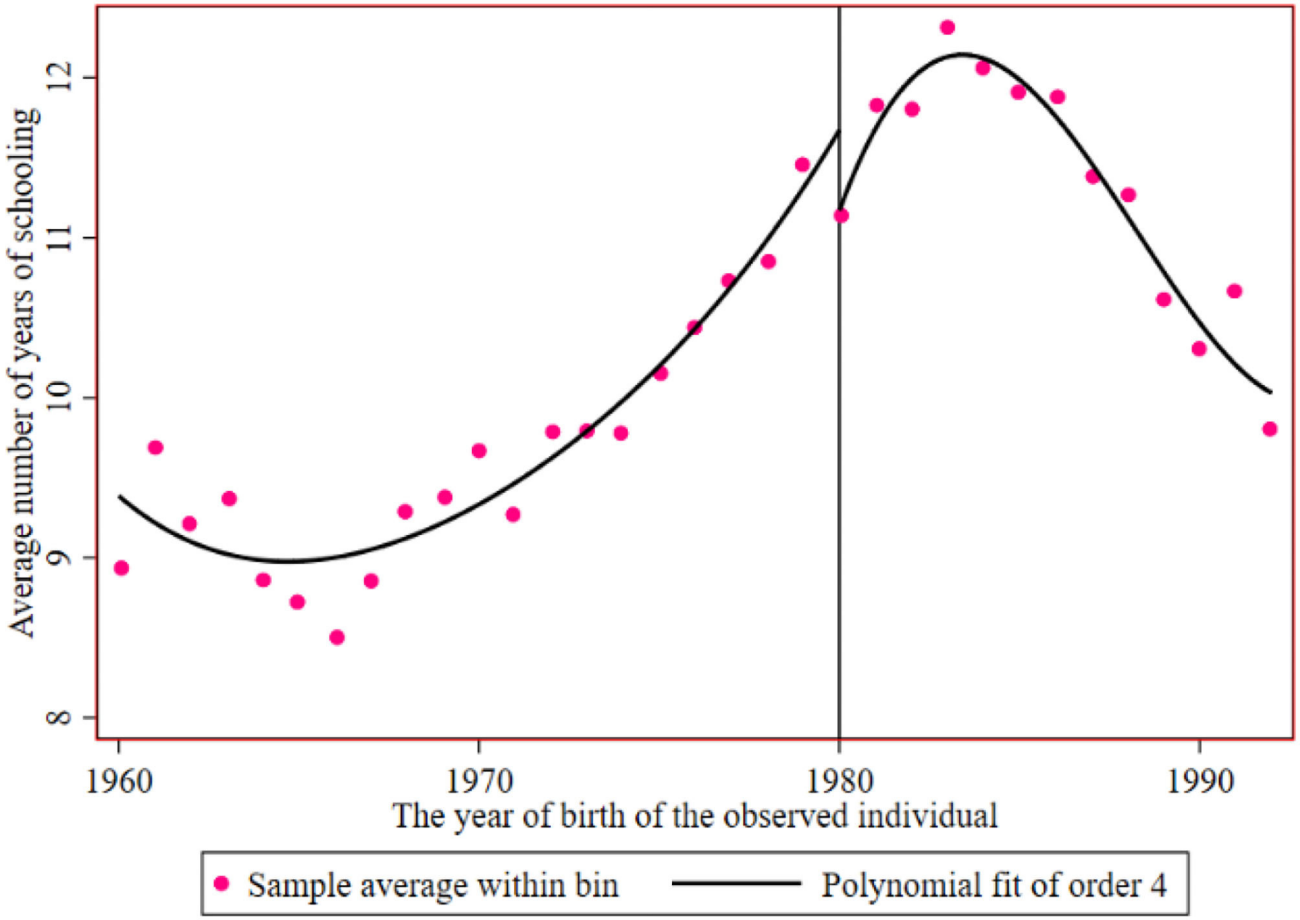
The educational breakpoint of rural residents before and after the implementation of the reproductive health policy.

**Figure 4:**
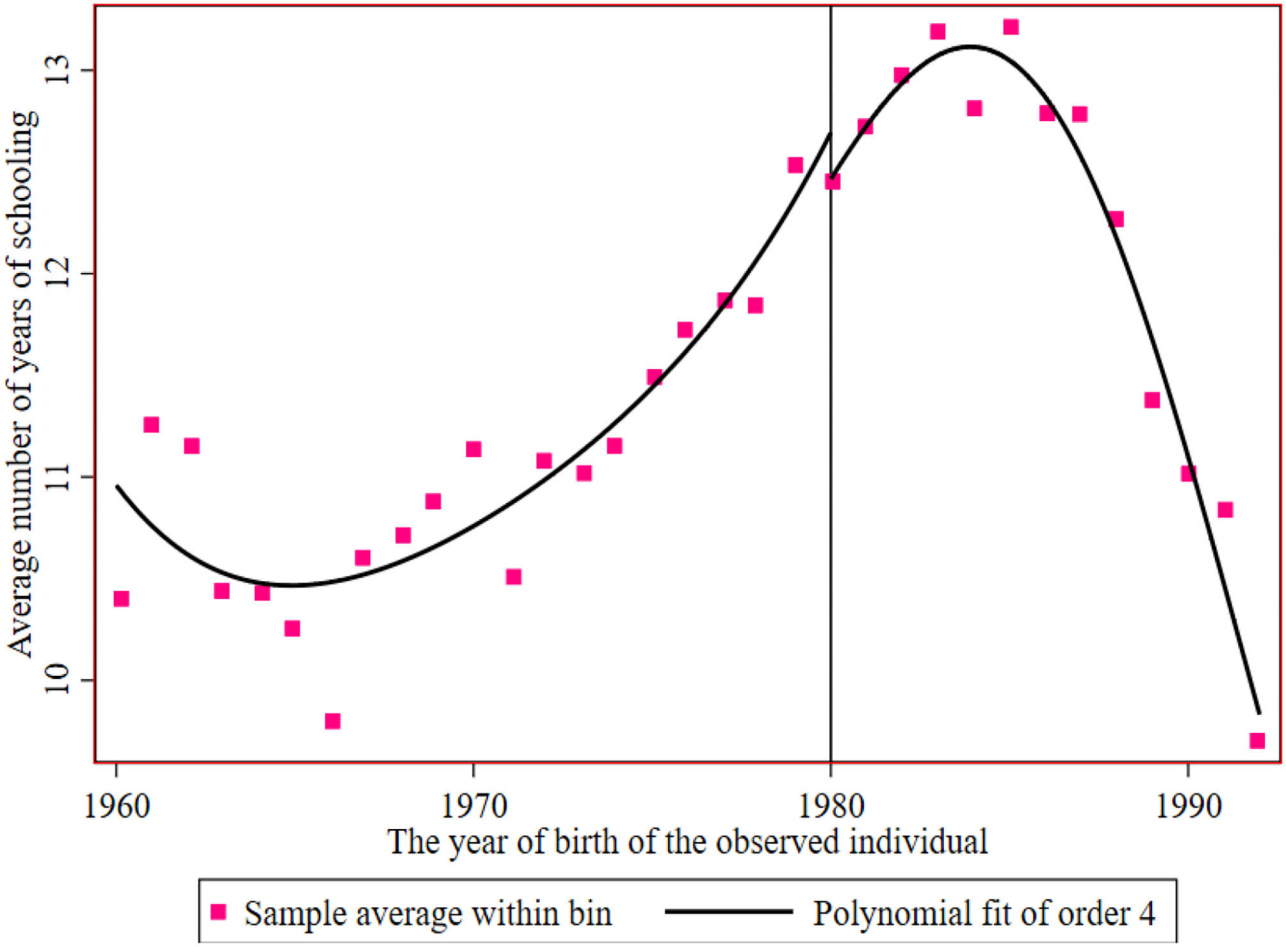
The educational breakpoint of urban residents before and after the implementation of the reproductive health policy.

Figures 3, 4 show the breakpoint of education of rural residents and urban residents before and after the implementation of the family planning policy. The number of years of education of the sample has a downwards trend under the influence of the family planning policy, with an intercept of approximately 11.6 years to 11.3 years. It can be said that the local treatment effect of the family planning policy is 0.3 years, but then it begins to rise. The reason for the decline may be that reproductive health policy was mainly encouraged at the beginning.

#### Regression Results Based on RD Estimation

Figures 2, 4 show that the logarithm of individual annual income and years of education of urban residents jump before and after the breakpoint of the family planning policy. The first is the estimated value without covariates, and the estimated results are shown in Table 6 (13). Second, the estimation results of covariates and other empty factors (sex, health status, ethnicity, marital status) were added, as shown in Column (14) of Table 6. The breakpoint regression estimation results obtained by nonparametric estimation using a rectangular kernel in Column (15) did not add covariates. In Column (16), the breakpoint regression estimation results obtained by nonparametric estimation using the rectangular kernel were added with covariates.

**Table 6:** Fuzzy RDD estimation results.

	**(13)**	**(14)**	**(15)**	**(16)**
*Panel A*:Logarithmic wage simplification equation				
*Policy*	0.0817***	0.0967***	0.0681***	0.0768***
	(0.0237)	(0.0240)	(0.0767)	(0.0789)
*R* ^2^	0.3127	0.3586	0.3127	0.3586
*Panel B*: Education simplified equation				
*Policy*	0.1537***	0.1349***	0.1348*	0.1349*
	(0.0032)	(0.0035)	0.0035	(0.0034)
*R* ^2^	0.1960	0.2118	0.1960	0.2118
*Panel C* : Logarithmic wage structural equation				
*Policy*	0.1286***	0.1216***	0.1226***	0.1334***
	(0.1334)	(0.3299)	(0.0608)	(0.1047)
Whether to include covariates	*No*	*Yes*	*No*	*Yes*
The critical value	In 1980	In 1980	In 1980	In 1980
To drive the variable	*Yes*	*Yes*	*Yes*	*Yes*
Control variables	*No*	*Yes*	*No*	*Yes*
Sample bottle	9497	9497	9497	9497

***, ^**^, ^*^ distribution means that statistics with significance levels of 1%, 5%, and 10% can be passed.

Table 6 shows the estimated results of fuzzy breakpoint regression. Panel A is based on the regression results of Mincer's income equation model, which shows that the implementation of the reproductive health policy has a significant impact on the logarithm of the annual total income of individuals. In the regression model, the impact of reproductive health policy on income is first negative and then positive, which is significant at least at the 10% level. The regression results of the control variables (sex, health status, ethnicity, and marital status) added in Column (16) show that the impact of the implementation of reproductive health policy on the logarithm of the annual total income of individuals is 0.0768, equivalent to 0.0817 standard deviations of logarithmic income. The equation results of the simplified results of education in Panel B show that the implementation of the family planning policy improves the number of years of education of individuals. There was no significant difference in the results of either the triangular kernel or rectangular kernel. When the model with control variables is added, the impact of the implementation of the family planning policy on the number of years of education of individuals is 0.1349 years. Panel C shows that the number of years of education has a positive impact on the logarithm of an individual's annual total income when instrumental variables are added. Model (13) contains the basic elements of Mincer's income equation model, and the estimated rate of return on education is 12.86%. When covariates are added, the estimated rate of return on education is 12.16%, and the results fluctuate by 0.6%. In the case of the rectangular kernel, the estimated educational return is 12.26%, and when covariates are added, the estimated educational return is 13.34%, with a 1.08% fluctuation in the result. The estimated result of Model (16) shows that the rate of return on education reaches 13.34%, and the estimated result of 2SLS shows that the rate of return of each additional year of education is 13.34%, which is significantly higher than the OLS estimate.

#### RD Validity Test

The validity of RD estimation results is easily affected by bandwidth selection. The smaller the bandwidth is, the more similar the influencing factors on both sides of the breakpoint are, and the less the influence of the missing variable on the dependent variable is. Therefore, the processing effect at the breakpoint can be estimated more accurately, and the estimation bias caused by endogeneity can be reduced.

**Figure 5:**
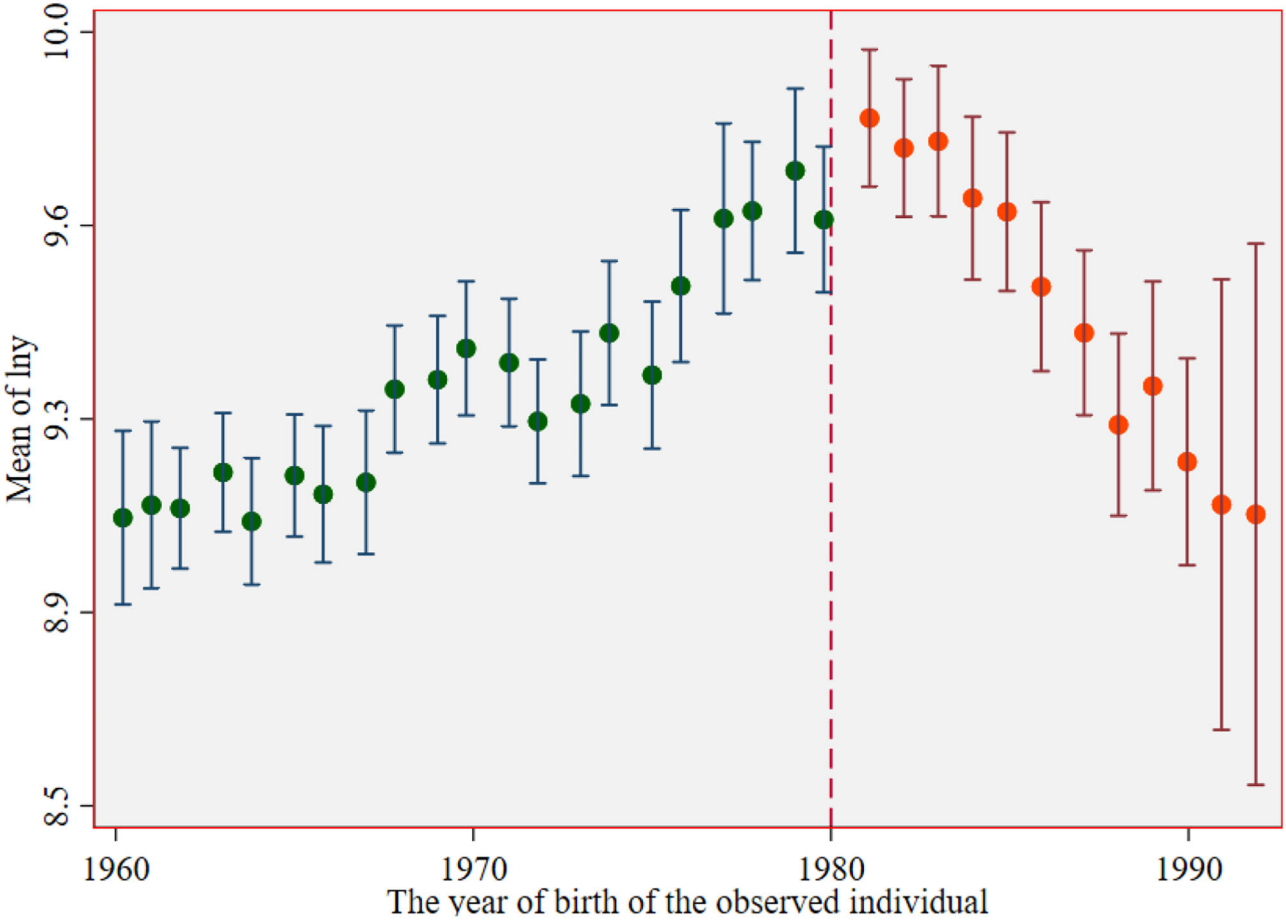
The change of density distribution of observed individuals before and after the breakpoint.

To test the effectiveness of RD, we need to test whether the individual can accurately manipulate the assignment. RD is not strict about the externality of the processing variable itself but assumes only that the sample individual cannot accurately manipulate the assignment variable. All relevant factors except the selected processing variables are smoothed at the breakpoint to avoid capturing the breakpoint effects of other factors. There are no potential manipulation problems with execution variables. The inherent advantage of RD regression is that the selected samples can be precisely positioned, and the density continuity performance of the execution variables is guaranteed. Figure 5 confirms that all the control variables we selected show continuous changes at the dividing line. Specifically, we require only that the policy shock that created the breakpoint cannot be precisely anticipated. To investigate whether there is a precise forecast of reproductive health policy, which in turn leads to accurate control of the number of children, we, according to the common practice in the literature, investigated assigned variable density distribution continuity (31): if the density distribution is discontinuous, it may exist for the precise manipulation of assigned children; otherwise, no precise control can be thought of. Following his method, Figure 5 shows the density distribution of the assigned variables. Both the density function curve and its confidence interval almost coincide at the breakpoint; that is, there is no significant jump. This indicates that the distribution of the birth time of sample individuals is continuous and smooth; that is, individuals do not accurately manipulate the assigned variables.

We need to check other control variables (including sex and whether there is a spouse, health, or ethnic group virtual variable) in the breakpoint density distribution continuity: if these control variables in the breakpoint are “jumping”, then the result of the variable “jump” cannot be fully explained by process variables, and causal inference loses effectiveness. To this end, we use the idea of local linear regression in RD to replace the original result variables with these control variables and obtain the change values of these covariables before and after breakpoints in Table 7. There is no obvious breakpoint near the truncation point of the four control variables, which meets the requirement of smoothness. We test the smoothness of the variables at the breakpoint, and the test results show that all the industry dummy variables fail the significance test at the breakpoint under the optimal window width setting (most broadband). In summary, these tests support the identification hypothesis that the occupational dummy variable is continuous at the cut-off point of the individual's birth year, suggesting that the breakpoint regression approach is appropriate.

**Table 7:** Continuity test for main control variables (based on RD regression).

**Variables**	**(17)**	**(18)**	**(19)**
	**50**	**100**	**200**
*minzu*	0.017	0.018	0.017
	(0.41)	(0.31)	(0.43)
*health*	−0.031	−0.034	−0.030
	(−1.22)	(−1.24)	(−1.23)
*hunyin*	−0.027	−0.026	−0.028
	(−1.17)	(−1.15)	(−1.14)
*gender*	0.007	0.008	0.005
	(0.11)	(0.14)	(0.13)
*lwald*	−0.215	−0.220	−0.218
	(−1.57)	(−1.47)	(−1.59)

*The estimates in this table do not control covariates. They are the estimated results obtained under the optimal bandwidth after replacing the RD result variables with covariables respectively. The z value is in parentheses*.

We examine whether the RD result is significant at other hypothetical threshold points, i.e., the falsification test: if the estimated result is significant at other threshold points, it is likely that the original RD result was not caused by a “jump” in the processing variable. For this reason, several other hypothetical breakpoints were randomly selected for regression within 1 year before and after 1980. Table 5 shows the RD estimation results of these hypothetical breakpoints. Local average treatment effect (LATE) estimates are not significant for these time points, which indicates that the significance of RD estimation results in Table 6 is not caused by a large sample size, thus further verifying the robustness and effectiveness of the second main conclusion.

The effectiveness of RD also depends on the smoothness hypothesis; that is, there is no significant jump between the two sides of the cut-off point for other control variables affecting income except years of education. They are observed evidence of physical health, sex, marital status, and ethnicity, respectively. The estimation method is the nonparametric local linear regression method proposed by Austin (32). Table 6 shows the test of the optimal broadband of the above four variables at the breakpoint, and the fitting effect is good. The above four control variables do not have obvious breakpoints near the truncation point, which meets the requirement of smoothness. In the case of the optimal window width, these test results support Hypothesis 1, which also indicates that the breakpoint regression method is appropriate, as seen in Table 8.

**Table 8:** Optimal broadband estimation results.

**lny**	**(17)**	**(18)**	**(19)**
*lwald*	0.1538 (0.1875)	0.82 (0.0120)	0.5213 (0.2136)
*lwald*50	0.0946 (0.1079)	0.88 (0.080)	0.3062 (0.1168)
*lwald*200	0.1097 (0.1084)	(0.012)	0.3222 (0.1027)

## Conclusions

This paper adopts breakpoint regression, baseline regression, and mediation effects to test the relationship between the reproductive health policy and the educational return rate. Tables 3, 5 show that reproductive health policy can promote the improvement of the educational return rate. First, the benchmark regression results of the whole sample show that the implementation of the family planning policy has significantly increased the number of years of education of the individual sample, and the rate of return on education for rural residents is only 5.31%, while that of urban residents is 11.72%, which may be due to the urban-rural income gap. Second, the mediating variables (consumption expenditure and housing area) are introduced to analyse the relationship between reproductive health policy and the rate of return on education. First, the implementation of reproductive health policy can affect changes in consumption expenditures and housing areas. Second, the breakpoint regression results show that the implementation of reproductive health policy significantly improves the rate of return on education for individuals in the sample. The years of education of the treatment group reached approximately 12.2 years, while that of the control group reached approximately 9.3 years, and the difference in years of education between the treatment group and the control group was 2.9 years. Using the breakpoint regression method, the educational return rate reached 7.8%. Some cities show very high returns on education, which indicates that the difference in educational return rate between regions is more reflected in cities than in provinces, and the difference between cities within provinces may have an expanding trend. From a practical point of view, the differences in economic development levels and the unbalanced allocation of educational resources are the reasons for the regional differences in educational returns.

The suggestions are made based on the regression results: The estimation results of this paper also reveal the investment and decision mechanism of Chinese household human capital to a certain extent, the intermediary effect indicates the importance of investment and consumption, and investment in education should be increased. China has taken comprehensive measures to achieve an appropriate fertility level, optimize the population structure, and promote long-term balanced population development. The difference in educational return rate stems from the difference in wage income of different workers, so it is very important to improve and perfect the labor market. First, further relaxation of restrictions on interprovincial and interregional labor mobility will help workers with higher education levels continue to migrate to regions with higher returns. As labor markets improve, the gap between the incomes of highly skilled and unskilled workers in low-return areas may widen, but this strengthens the incentive to invest in education in those areas. In the long run, a sound labor market is conducive to economic growth and orderly and reasonable flow of human capital, which can maximize the allocation of human capital and lay an economic foundation for the supply and investment of educational resources. Second, in the process of promoting new urbanization, we should fundamentally break the urban-rural separation and encourage the rational flow of urban and rural populations, which is particularly important for the human capital investment of rural residents. At present, China's national conditions have gradually deteriorated the aging of the population. Considering the economy, education, reproductive health policy, and other factors affecting health, China should improve the comprehensive protection of water. Second, education should keep up with the speed of social and economic development. When implementing fertility policies, attention should be given to the adjustment of educational preferences and the alleviation of family investment constraints on children, such as the construction of credit markets in underdeveloped areas and the establishment of low-interest or interest-free student loan mechanisms, and consideration should be given to regional differences in the impact of various factors on population health. The promotion policy of differentiated population healthy reproductive health policy should be formulated accordingly. By improving the social security system, deepening the reform of the medical and health system and advocating a healthy lifestyle, we can promote the continuous improvement in the health level of the population, which is conducive to improving the rate of return on education. Third, implementing the concept of gender equality education, implementing more equitable education policies, vigorously improving the education level and improving the health literacy of residents will further pave the way for China to improve the rate of return on education, which will be conducive to the adjustment of China's current population policy.

## Data Availability Statement

The original contributions presented in the study are included in the article/supplementary material, further inquiries can be directed to the corresponding author.

## Author Contributions

QG contributed to the conceptualization and methodology. X-XR wrote and edited the manuscript. W-LY is responsible for the software, writing the original draft, and reviewing. A-HW wrote and reviewed the manuscript. W-HZ contributed to the software and data preparation. All authors contributed to the article and approved the submitted version.

## Funding

This research is partly supported by Philosophy and Social Sciences Fund of Guangxi Province (21CJY013), Education Planning Project of Guangxi Province (2021A043), Guangxi Higher Education Undergraduate Teaching Reform Project (2020JGA236), Philosophy and Social Sciences Fund of Guangxi Province (20CJY001), and Philosophy and Social Sciences Fund of Guangxi Province (20BTJ001).

## Conflict of Interest

The authors declare that the research was conducted in the absence of any commercial or financial relationships that could be construed as a potential conflict of interest.

## Publisher's Note

All claims expressed in this article are solely those of the authors and do not necessarily represent those of their affiliated organizations, or those of the publisher, the editors and the reviewers. Any product that may be evaluated in this article, or claim that may be made by its manufacturer, is not guaranteed or endorsed by the publisher.

## References

[1] Turčínková J Stávková J. Does the attained level of education affect the income situation of households? Procedia Soc. (2012) 55:1036–42 10.1016/j.sbspro.2012.09.595

[2] Han S Mulligan CB. Human capital, heterogeneity and estimated degrees of intergenerational mobility. Econ J. (2001) 111:207–43 10.1111/1468-0297.00606

[3] Kalwij A. Estimating the economic return to schooling on the basis of panel data. Appl Econ. (2000) 32:61–71 10.1080/000368400322985

[4] Card D. Chapter 30 The causal effect of education on earnings. Handbook of Labor Economics. (1999) 3:1. 10.1016/S1573-4463(99)03011-430212146

[5] Jamison Dean T Jacques VG. Education and earnings in the People's Republic of China. Pergamon. (1987) 6:161–6. 10.1016/0272-7757(87)90049-5

[6] Li H Liu PW Zhang J. Estimating returns to education using twins in urban China. J Dev Eco. (2011) 97:494–504. 10.1016/j.jdeveco.2011.05.009

[7] Siphambe H. Rates of return to education in Botswana: results from the 2002/2003 household income and expenditure survey data set. S. Afr. J. Econ. (2008) 76:641–51. 10.1111/j.1813-6982.2008.00211.x

[8] Knight J Song L. Increasing urban wage inequality in China. Econ Transit. (2003) 11:597–619. 10.1111/j.0967-0750.2003.00168.x

[9] Yang Y. A research on the difference of education returns between urban and rural areas. Open J Soc Sci. (2017) 5:59–67. 10.4236/jss.2017.54006

[10] Lounkaew K. Explaining urban–rural differences in educational achievement in Thailand: Evidence from PISA literacy data. Econ Educ Rev. (2013) 37:213–25. 10.1016/j.econedurev.2013.09.003

[11] Rouse CE. Further estimates of the economic return to schooling from a new sample of twins. Econ Educ Rev. (1999) 18:1157–73. 10.1016/S0272-7757(98)00038-7

[12] Heckman PE. School restructuring in practice: reckoning with the culture of school. Int J Educ Reform. (1993) 2:263–72. 10.1177/105678799300200305

[13] Chen G Hamori S. Economic returns to schooling in urban China: OLS and the instrumental variables approach China. Eco Rev. (2009) 20:143–52. 10.1016/j.chieco.2009.01.003

[14] Yakita A. Fertility and education decisions and child-care policy effects in a Nash-bargaining family model. J Populat Eco. (2018) 31:37–50. 10.1007/s00148-017-0675-7

[15] Yang Z. Research and implementation of basic education resource retrieval strategy based on the relationship among knowledge points. MATEC Web Conf . (2018) 173:30–57. 10.1051/matecconf/201817303057

[16] Sayers J. The environmental education television project for China. Continuum. (2003) 17:273–6. 10.1080/10304310302736

[17] Liao J Zhao J. Rate of returns to education of persons with disabilities in rural china proceedings of the 2013. International Conference on Applied Social Science Research (ICASSR-2013). (2013) 54–5. 10.2991/icassr.2013.60

[18] Kai-Hua W Chi-Wei S Muhammad U. Geopolitical risk and crude oil security: A Chinese perspective. Energy. (2021) 219:896–907. 10.1016/J.ENERGY.2020.119555

[19] Lee J Ihm J. Gender difference in returns to education independent of gender wage gap in Korea^*^. Asian Econ. J. (2020) 34:213–32. 10.1111/asej.12209

[20] Chi-Wei S Khalid K Muhammad U Weike Z. Does renewable energy redefine geopolitical risks? Energy Policy. (2021) 158:158–60. 10.1016/J.ENPOL.2021.112566

[21] Peter G David SE. Early academic performance, grade repetition, and school attainment in senegal: a panel data analysis. Narnia. (2010) 24:93–120. 10.1093/wber/lhp023

[22] Black SE Devereux PJ Salvanes KG. The more the merrier? the effect of family size and birth order on children's education. Q J Econ. (2005) 120:669–700. 10.1093/qje/120.2.669

[23] Song S Wang W. Testing the only-child advantage in cognitive development in the context of china's one-child policy. Popul Res Policy Rev. (2019) 38:841–67. 10.1007/s11113-019-09556-9

[24] Ran T Chi-Wei S Bushra N Abbas RSK. Can Fintech development pave the way for a transition towards low-carbon economy: A global perspective Technological. Technol Forecast Soc. (2022) 174:165–75. 10.1016/J.TECHFORE.2021.121278

[25] Carolina B Edgar D Velasco Bárbara EI Juan R. Malnutrition prevalence among children and women of reproductive age in Mexico by wealth, education level, urban/rural area and indigenous ethnicity. Public Health Nutr. (2020) s77–s88. 10.1017/S1368980019004725 PMC10201355 32148210

[26] Blaug M. Schooling, experience and earnings. Jacob Mincer Mark Blaug. (1976) 25:166–71. 10.1086/450936

[27] Manohar S. Mediation effect of service quality between service innovation and customer word-of-mouth in Indian higher education system. Int J of Business Excellence. (2018) 16:127–48 10.1504/IJBEX.2018.094701

[28] Abell ML Fraser MW Galinsky MJ. Early intervention for aggressive behavior in childhood: a pilot study of a multi-component intervention with elementary school children and their families. J Fam Soc Work. (2001) 6:19–37. 10.1300/J039v06n04_03

[29] Qin M Su C-W Tao R. BitCoin: A new basket for eggs? Econ. Model. (2020) 9:896–907. 10.1016/j.econmod.2020.02.031

[30] Malik I Dewancker B. Identification of population growth and distribution, based on urban zone functions. Multidisciplinary Digital Publishing Institute. (2018) 10:930. 10.3390/su10040930

[31] McCrary J. Manipulation of the running variable in the regression discontinuity design: A density test. J Economet. (2007) 142:698–714. 10.1016/j.jeconom.2007.05.005

[32] Nichols A. Causal inference with observational data. Stata J. (2007) 7:507–41. 10.1177/1536867X0700700403

